# Identification of QTLs for root color and carotenoid contents in Japanese orange carrot F_2_ populations

**DOI:** 10.1038/s41598-022-11544-7

**Published:** 2022-05-16

**Authors:** Taeko Shibaya, Chika Kuroda, Hisano Tsuruoka, Chiharu Minami, Akiko Obara, Shinobu Nakayama, Yoshie Kishida, Takayoshi Fujii, Sachiko Isobe

**Affiliations:** 1 Fujii Seed Co. Ltd., Fujii Seed, 2-12-38 Juso-higashi, Yodogawa-ku, Osaka, 532-0023 Japan; 2 Kazusa DNA Research Institute, Kisarazu, Chiba 292-0818 Japan

**Keywords:** Genetics, Plant sciences

## Abstract

Carrot is a major source of provitamin A in a human diet. Two of the most important traits for carrot breeding are carotenoid contents and root color. To examine genomic regions related to these traits and develop DNA markers for carrot breeding, we performed an association analysis based on a general liner model using genome-wide single nucleotide polymorphism (SNPs) in two F_2_ populations, both derived from crosses of orange root carrots bred in Japan. The analysis revealed 21 significant quantitative trait loci (QTLs). To validate the detection of the QTLs, we also performed a QTL analysis based on a composite interval mapping of these populations and detected 32 QTLs. Eleven of the QTLs were detected by both the association and QTL analyses. The physical position of some QTLs suggested two possible candidate genes, an *Orange* (*Or*) gene for visual color evaluation, and the α- and β-carotene contents and a *chromoplast-specific lycopene β-cyclase* (*CYC-B*) gene for the β/α carotene ratio. A KASP marker developed on the *Or* distinguished a quantitative color difference in a different, related breeding line. The detected QTLs and the DNA marker will contribute to carrot breeding and the understanding of carotenoid biosynthesis and accumulation in orange carrots.

## Introduction

Carrot (*Daucus carota* L.), a major source of provitamin A carotenes in the human diet, is consumed worldwide^1^. Carrots accumulate abundant carotenoids in their taproots, and these carotenoids (which are responsible for the orange pigmentation in the carrot roots) are thought to provide health benefits^2^. A variety of colors has been observed in carrot taproots, including orange, white, yellow, red, and purple. Quantitative trait loci (QTL) analyses and association studies for carrots' root color and carotenoid contents have been performed in several populations, and important and useful QTLs have been reported^3–6^. These studies used populations derived from crosses between accessions showing clearly different root colors such as orange and white^3–5^ and orange and dark orange^4^, and other studies used inter-crossed populations derived from white, yellow, red, and orange carrots^6^.

**Figure 1:**
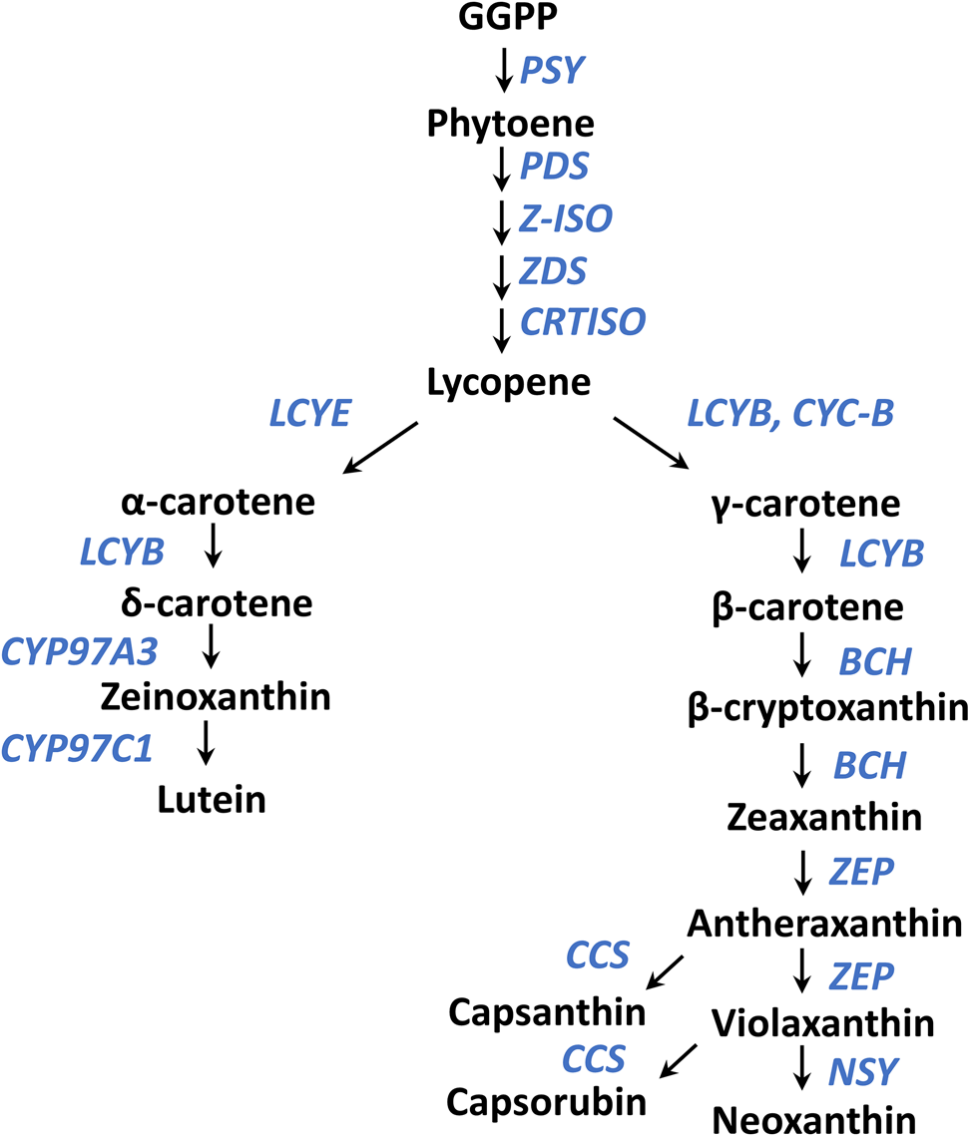
Carotenoid biosynthesis pathways. The carotenoid biosynthesis pathway is shown in *black*, with carotenoid biosynthesis genes indicated in *blue*. Figure compiled and summarized from Stanley et al.^8^ and Al-Babili et al.^9^. *PSY*, *phytoene synthase*; *PDS*, *phytoene desaturase*; *Z-ISO*, *ζ-carotene isomerase*; *ZDS*, *ζ-carotene desaturase*; *CRTISO*, *carotene isomerase*; *LCYE*, *lycopene ε-cyclase*; *LCYB*, *lycopene β-cyclase*; *CYP97A3*, *cytochrome P450-type β-hydroxylase*; *CYP97C1*, *cytochrome P450-type ε-hydroxylase*; *CYC-B*, *chromoplast-specific lycopene β-cyclase*; *BCH*, *β-carotene hydroxylase*; *ZEP*, *zeaxanthin epoxidase*; *NSY*, *neoxanthin synthase*; *CCS*, *capsanthin-capsorubin synthase*.

Carotenoid biosynthesis is well established, and a highly conserved carotenoid biosynthesis pathway has been characterized in many plant species (Fig. 1)^7–9^. In carrot, several carotenoid biosynthetic genes have been mapped^3^, and the released carrot whole-genome sequences showed orthologous and homologous genes involved in the carotenoid biosynthesis pathway^10,11^. Several genes involved in carotenoid biosynthesis and accumulation in carrot have also been identified. An ortholog of carotene hydroxylase CYP97A3 in the carotenoid biosynthesis pathway has been identified in carrot; it controls the α-carotene, total carotenoid contents, and the α/β carotene ratio^12^. A candidate gene association study of the carotenoid biosynthesis pathway revealed associations between the total carotenoid and β-carotene contents and the genes *zeaxanthin epoxidase* (*ZEP*), *phytoene desaturase* (*PDS*), and *carotenoid isomerase* (*CRTISO*), between the α-carotene content and the genes *CRTISO* and *plastid terminal oxidase* (*PTOX*), and between color components and the gene *ZEP*^6^.

It was also reported that not only genes in the carotenoid biosynthesis pathway but also genes that have other functions considerably affect carotenoid contents. *Y* and *Y*_*2*_ loci account for most of the color differences of orange, yellow, and white carrot roots^13^. The *Y* gene has been identified, and this gene has been hypothesized to regulate photosystem development and functional processes, including photomorphogenesis and root de-etiolation^10^. The *Y2* locus has been mapped to an approx. 650-kb genomic region; in addition, no annotated gene involved in the carotenoid biosynthesis pathway was located within the candidate region^14^. An *Orange* (*Or*) gene, which was first identified in cauliflower and accounted for an abnormally elevated β-carotene accumulation^15^, was identified in carrot and is associated with the presence of carotenoid in carrot^16^. However, the genes, polymorphisms, and QTLs involved in carotenoid biosynthesis and the carotenoid accumulation that cause quantitative differences in root color and carotenoids are not fully understood, especially within the orange carrots.

In Japan, consumers prefer a bright orange root color for carrots, and a cultivar showing uniform root colors is popular. There are accessions showing quantitative color differences in bright orange roots, and breeders in Japan have selected the best 'bright orange' and uniform color among the accessions that have bright orange roots. DNA markers that can be used to distinguish quantitative differences within bright orange color have thus been sought in Japanese carrot breeding. Toward this goal, there has been no study using populations derived from a cross between orange root carrots with quantitative color differences, but the recent release of whole genome sequences of carrot has made it easier to analyze whole-genome constitutions with high marker density, even in the populations derived from genetically close orange carrots^10,11^.

In the present study, we developed two F_2_ populations that have a common parent. Both populations were derived from crosses between orange-root parents. We performed an association and QTL analyses to detect QTLs that cause quantitative but important differences in the root color and carotenoid contents within carrots with orange root color.

## Methods

### Plant materials

**Figure 2:**
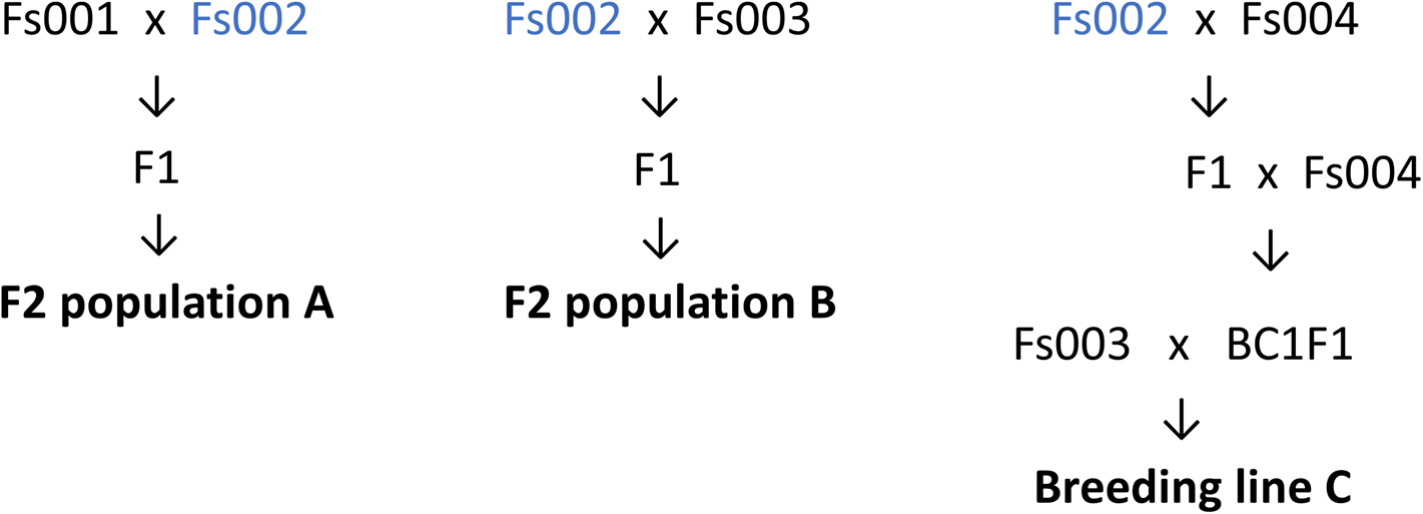
Lineage images of the plant materials, F_2_ populations A and B, and breeding line C. Fs002 was used as a common breeding material.

We developed two F_2_ populations (A and B) using orange-colored carrot plants bred by a Japanese seed company, Fujii Seed (Osaka, Japan). Population A was derived from a cross between Fs001 and Fs002, and population B was derived from a cross between Fs002 and Fs003 (Fig. 2). Fs002 was the pollen parent for F_2_ population A and the seed parent for F_2_ population B. Plants of F_2_ populations A (n = 146) and B (n = 136) were cultivated from mid-February to early June 2018 in a natural field at Narashino, Chiba, Japan, and used for DNA extraction and the visual evaluation of root colors. Roots of population A were also used for the quantification of carotenoid content by high-performance liquid chromatography (HPLC) and the measurement of color components.

To examine a developed DNA marker on *Or* gene, we also used breeding line C, which was bred by Fujii Seed. This line was developed by using Fs002 as one of the breeding materials (Fig. 2). Breeding line C was cultivated from the end of March to early July 2017 in a natural field at Oirase, Aomori, Japan, and 40 plants were used for DNA extraction and the visual evaluation of root colors.

Experimental research and field studies on plant materials comply with relevant institutional, national, and international guidelines and legislation.

### Visual evaluation of root colors and evaluation of color components

The visual evaluation of root colors was performed by two experienced breeders at Fujii Seed. The root colors were visually evaluated to ten grades of orange darkness in F_2_ population A, and to seven grades in F_2_ population B, and to three grades in breeding line C. In the F_2_ population A, color components (L*, a* and b*) were measured with a spectrocolorimeter (model CM2600d, Minolta, Tokyo) equipped with a 5-mm measuring area. The color components L*, a* and b* are components of CIELAB (also known as or CIE L*a*b*) color space. The color component L* represents perceptual lightness and defines black as 0 and white as 100. The color component a* represents the green–red opponent colors, with negative values toward green and positive values toward red. The color component b* represents the blue-yellow opponents, with negative values toward blue and positive toward yellow. The surface of the middle part of washed carrot root was measured three times, and the average values were used for phenotypic data.

### Quantification of carotenoid contents (α-carotene, β-carotene, and lutein) by HPLC

Carrot root surface, i.e., approx. 1–2 mm of epidermis and outer phloem in the middle of roots was cut and collected. The collected samples were immediately frozen in liquid nitrogen and stored at − 80 °C. Root epidermis and outer phloem was used for HPLC because the visual and color component evaluations were performed on the carrot root surface. The extraction for HPLC was performed as described^6^ with a scale-down and some modifications. Frozen samples were crushed into a powdery status with a tube mill control (S001, IKA, Staufen, Germany). Extraction was done on approx. 50 mg (50 mg ± 5%) of crushed frozen material to which 50 µL of b-apo-8'-carotenal at 5 µg/mL was first added as an internal standard. Samples were mixed with 600 µL of MgCO_3_ 0.57%, 3,5-di-tert-butyl-4-hydroxytoluene (BHT) 0.1% in methanol, then vortexed, and mixed with 600 µL of 0.1% BHT-containing chloroform. After 10 times of vertical mixing and incubation for 15 min in darkness at 4 °C, 600 µL of ultrapure water was added, and samples were centrifuged at 236 g for 10 min. Next, 400 µL from the lower layer was concentrated under vacuum evaporation, and the dry extract was dissolved in 50 µL of acetone containing 0.1% BHT. Samples were kept at 4 °C and protected from direct light during the entire procedure.

The carotenoid quantification was done on an Ultimate 3000 HPLC system coupled with a diode array detector (Thermo Fisher Scientific, Waltham, MA, USA) according to the manufacturer's instruction with slight modifications. Carotenoids were separated on an Acclaim C30 column (150 × 2.1 mm, 3 µm, Thermo Fisher Scientific). The mobile phases were acetonitrile as eluent A, methanol/acetic ether (1:1, v/v) as eluent B, and 10 mM formic acid (pH 3.0) as eluent C. The elution program was as follows: the proportions of solvent A, B and C were 85% A, 14.5% B, and 0.5% C at 0–2 min; 85%–44.5% A, 14.5%–55% B, and 0.5% C at 2–7 min; 44.5% A, 55% B, and 0.5% C at 7–21 min; and returned to the initial conditions (85% A, 14.5% B, and 0.5% C) at 21.1–28.5 min. The flow rate was 0.4 mL/min. The injection volume of the filtered sample by a 0.22-µm PTFE membrane filter was 3.9 µL. Analytes were detected by a photodiode array detector at 450 nm. The data were analyzed using Chromeleon 7 software (Thermo Fisher Scientific) based on internal calibration using b-apo-8'-carotenal and the extraction yield.

### Double-digest restriction site-associated DNA sequencing (ddRAD-seq)

Total genomic DNA was extracted from young leaves of carrot plants with the DNeasy Plant Mini Kit (Qiagen, Hilden, Germany). A double-digest restriction site-associated DNA sequencing (ddRAD-seq) analysis was performed as described^17^ with the restriction enzymes *Pst*I and *Msp*I. The ddRAD-seq libraries were constructed and sequenced on a HiSeq 4000 platform (Illumina, San Diego, CA) in paired-end 101-nucleiotide (nt) mode as described^17^. Primary data processing such as deleting low-quality bases and trimming adapters, mapping onto reference genome, and filtering single-nucleotide polymorphisms (SNPs) to obtain high-confidence SNPs were performed as described with some modifications^18^. In brief, low-quality sequences were removed and adapters were trimmed using PRINSEQ (ver. 0.20.4)^19^ and fastx_clipper in the FASTX-Toolkit (ver. 0.0.13) (http://hannonlab.cshl.edu/fastx_toolkit). The filtered reads were mapped onto carrot genome Daucus carota v2.0^10^ with Bowtie 2 (ver. 2.1.0; parameters: –minins 100 –no-mixed)^20^. The resultant sequence alignment/map format (SAM) files were converted to binary sequence alignment/map format (BAM) files and subjected to SNP calling using the mpileup option of SAMtools (ver. 0.1.19; parameters: default)^21^ to yield a variant call format (VCF) file including SNP information. The VCF files were filtered with VCFtools (ver. 0.1.14)^22^. The parameters for VCFtools were as follows: –maf 0.05 –max-alleles 2 –min-alleles 2 –minDP 5 –minQ 999 –maxmissing 0.95 –remove-indels.

### QTL detection by association and QTL analyses

For the association analysis, we detected QTLs based on a general linear model (GLM) by using trait analysis by association, evolution, and linkage (TASSEL) ver. 5.2.40^23^. The thresholds for the association were set as 1.6 × 10^−5^ (= 0.05/3159) and 2.6 × 10^−5^ (= 0.05/1901) at a significance level of 5% after Bonferroni correction in F_2_ populations A and B, respectively.

Linkage maps for the QTL analysis were constructed using Lep-MAP3^24^ from filtered VCF files. A filtering module was used for the marker quality filtering set as dataTolerance = 0.001 to exclude segregation distorted markers. The Separate Chromosomes module was set as lodLimit = 8 for F_2_ population A and lodLimid = 20 for F_2_ population B following Join Singles and Order Markers modules set as informativeMask = 123 and sexAveraged = 1. QTL analyses were performed using composite interval mapping implemented by the Zmapqtl program (model 6) provided in ver. 2.5 of Windows QTL Cartographer^25^. Genome-wide threshold values (α = 0.05) were used to detect putative QTLs based on the results of 1,000 permutations.

### Sanger sequencing of candidate genes

For the comparison of the genomic sequences of possible candidate genes between parental plants in F_2_ populations A and B, we performed Sanger sequencing from the start codon to the stop codon on the genes. The primers used in the Sanger sequencing are listed in Supplementary Table S1.

### SNP genotyping with KASP marker

KASP marker, which genotypes an SNP on *Or* gene in this study, was developed and performed according to the manufacturer's instructions (Biosearch Technologies, Novato, CA).

### Phylogenetic analysis

The sequences of LCYE, LCYB, CYC-B, NSY, and CCS in several reported plant species such as *Solanum lycopersicum*, *Carica papaya*, *Citrus sinensis*, *Capsicum annuum*, and *Lillium lancifolium*^26–29^, *Arabidopsis*^7^, and carrot^30^ were obtained from the public databases NCBI (http://www.ncbi.nlm.nih.gov) and Phytozome (https://phytozome.jgi.doe.gov/pz/portal.html#). We used CLUSTALW^31^ to align the amino acid sequences and constructed the phylogenetic tree by using the neighbor-joining method^32^ provided by MEGA X^33^.

## Results

### Association analysis for the visual evaluation of root color and evaluations of color components and carotene contents in roots of F_2_ populations A and B

**Figure 3:**
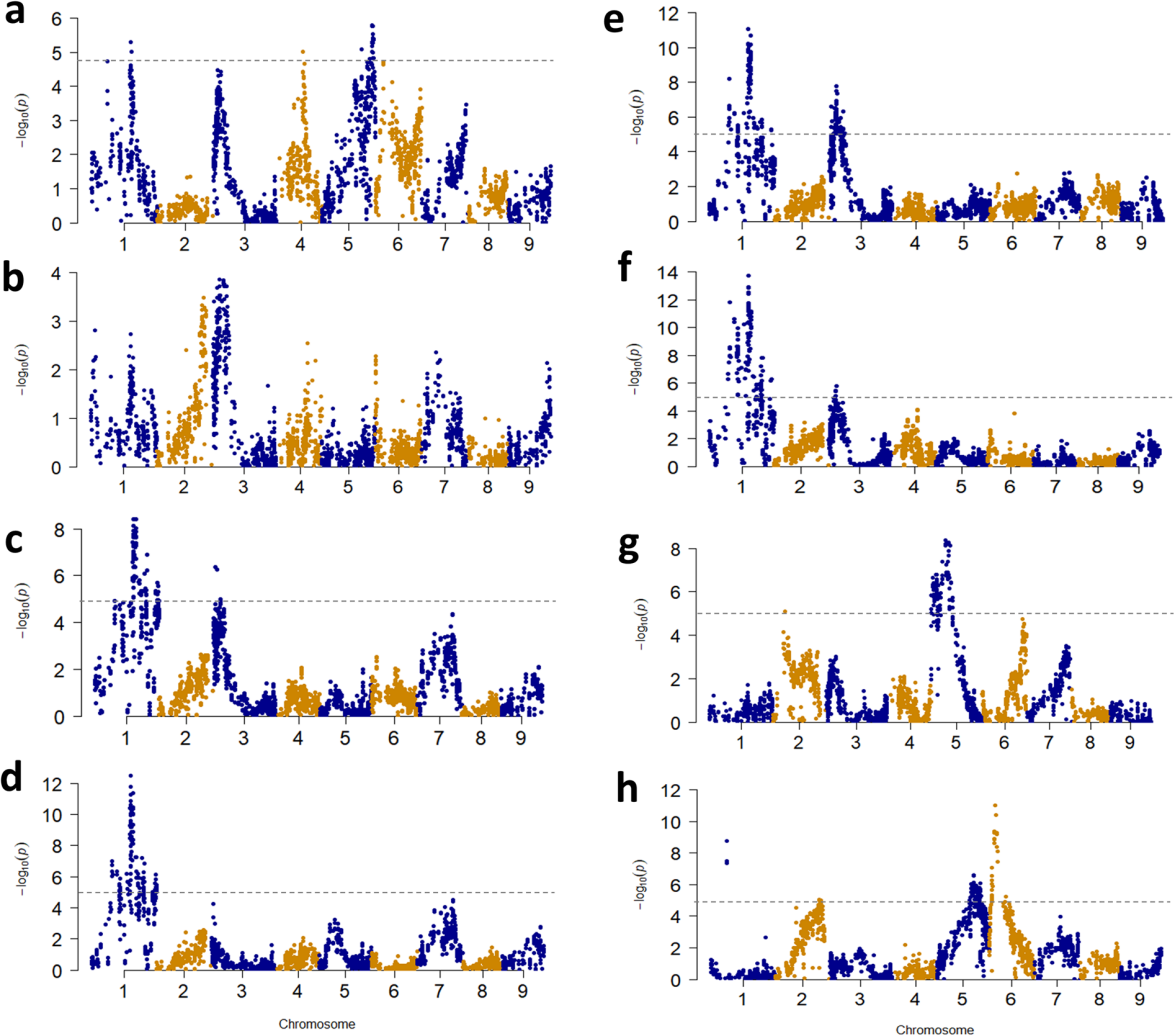
Manhattan plots for carrot taproot color in F_2_ population A. Plots for the visual evaluation (**a**), L* (**b**), a* (**c**), b* (**d**), α-carotene content (**e**), β-carotene content (**f**), lutein content (**g**), and the β/α-carotene ratio (**h**). *Horizonal line* indicates the Bonferroni correction (0.05).

F_2_ populations A and B both showed a normal distribution in all root color evaluations (Suppl. Fig. S1), suggesting polygenic inheritance in carrot root color. The ddRAD-seq analysis detected 3,159 and 1,901 high-confidence SNPs in F_2_ populations A and B, respectively. The associations were investigated using these genotypic data and values from the visual evaluation and evaluations of the color components and carotene contents in the carrot roots. In F_2_ population A, significant associations were detected for the visual evaluation of root color (Fig. 3a); color components a* (Fig. 3c) and b* (Fig. 3d); α-carotene (Fig. 3e), β-carotene (Fig. 3f), and lutein contents (Fig. 3g); and the β/α-carotene ratio (Fig. 3h) in root (Table 1). No significant associations were detected for color component L* (Fig. 3b).

**Table 1:** Significant associations for carrot root color identified by association analysis in F_2_ population A and B.

Population	Trait	Physical position^a^		− log10P
		Chr	bp	
A	Visual evaluation	1	30,782,032	5.31
		4	20,589,731	5.01
		5	39,247,011	5.80
		6	6,440,506	4.72
	a*	1	32,693,618	8.42
		3	1,689,065	6.37
	b*	1	31,112,534	12.49
	α-carotene	1	15,832,013	8.22
		1	30,704,558	11.08
		3	5,849,853	7.77
	β-carotene	1	15,832,013	11.82
		1	30,704,558	13.72
		3	6,455,250	5.79
	Lutein	2	10,281,204	5.09
		5	11,540,998	8.38
		6	32,679,396	4.75
	β/α-carotene ratio	1	12,574,447	8.78
		2	34,903,955	5.04
		5	29,337,124	6.63
		6	4,640,387	11.02
B	Visual evaluation	3	5,419,538	9.69

^a^Physical position of the SNP showing the most significant association in the association analysis based on carrot genome Daucus carota v2.0 (10).

**Figure 4:**
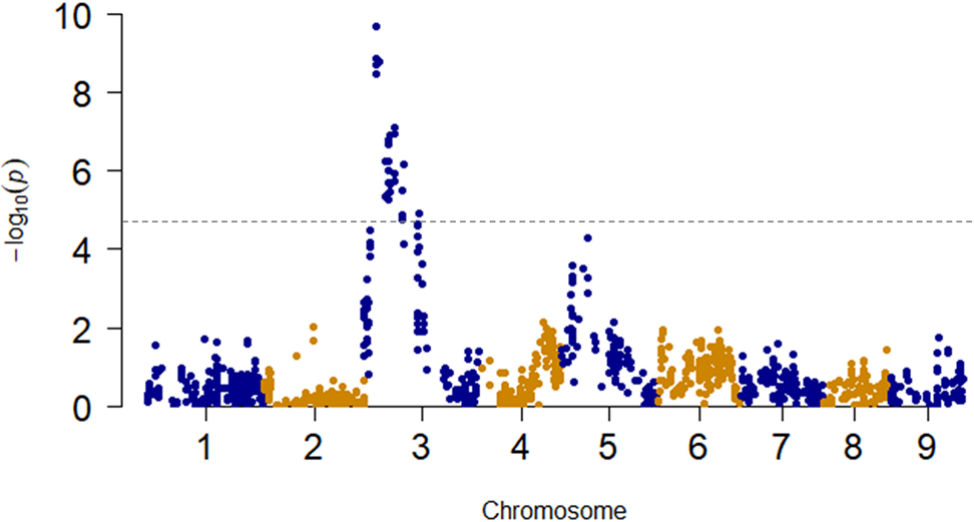
Manhattan plots for carrot taproot color in F_2_ population B. Plots for the visual evaluation of carrot root color. *Horizonal line:* Bonferroni correction (0.05).

The associations for visual evaluation, color components a* and b*, and α- and β-carotene contents on chromosome 1 were detected at close physical positions, and the highest associations were detected at a physical position around 31 Mb (Fig. 3, Table 1), suggesting that these associations are caused by an identical QTL. The physical positions of the associations for the α- and β-carotene contents on chromosome 3 were close, and the highest associations were detected at a physical position around 6 Mb (Fig. 3, Table 1). An association detected in population B for visual color evaluation on chromosome 3 showed the highest association at physical position 5.4 Mb, and this physical position was similar to those of the associations detected in population A for α- and β-carotene contents (Figs. 3, 4, Table 1). These results suggest that the associations would be caused by an identical QTL. Interestingly, the association detected on chromosome 5 (showing the highest association for visual evaluation in F_2_ population A) was not detected in any other evaluations (Fig. 3, Table 1).

### QTL analysis for the visual evaluation of root color and evaluations of color components and carotene contents in roots of F_2_ populations A and B

To examine the validity of QTLs detected by the association analysis, we also performed a QTL analysis constructing linkage maps. Marker segregation distortion in the two F_2_ populations was examined by Chi-squared test against the expected 1:2:1 ratio. In populations A and B, 2,370 markers of a total of 3,159 markers and 1,442 markers of a total of 1,901 markers segregated at a 1:2:1 ratio (*P* < 0.01), respectively. We thus analyzed these two populations as F_2_ generations. With the use of Lep-MAP3^24^, with segregation distorted markers excluded by the filtering module and non-grouped markers, 2,481 markers were mapped onto nine carrot chromosomes in F_2_ population A, and 1,586 markers were mapped onto seven carrot chromosomes and four linkage groups in F_2_ population B (Suppl. Tables S2–S4, Suppl. Figs. S2, S3).

The QTL analysis detected 31 QTLs for all traits examined in F_2_ population A (Table 2, Suppl. Fig. S4). Based on the physical position of the nearest marker, 11 QTLs were detected on the regions corresponding to the results of the association analysis (within 2 Mb) (Table 2). The QTL at a physical position around 31 Mb on chromosome 1 was detected for visual evaluation, color components a* and b*, and α- and β-carotene contents. This QTL was also detected by the association analysis for the same traits (Table 1, Fig. 3).

**Table 2:** QTLs for carrot taproot color in F_2_ population A and B detected by QTL analysis.

Pop^a^	Trait	Nearest marker^b^	Genetic position (cM)	LOD^c^	Additive effect^d^	PVE (%)^e^	AS^f^
A	Visual evaluation	Chr1_22103259	42.22	4.80	− 2.4332	9.31	n.d
		Chr1_30090002	53.30	6.73	0.5495	14.93	d
		Chr3_6731021	11.38	9.06	1.1171	18.86	n.d
		Chr5_39489714	64.85	4.08	− 0.7388	7.15	d
	L*	Chr1_3652405	13.06	3.57	0.8620	7.88	n.d
		Chr3_6731021	11.38	4.07	0.0778	11.07	n.d
	a*	Chr1_30782032	53.99	11.88	− 1.4784	23.38	d
		Chr3_4136215	7.94	5.81	0.6945	9.74	n.d
		Chr7_13266188	27.23	4.05	− 1.0896	6.91	n.d
	b*	Chr1_30782032	53.99	9.34	− 1.2117	17.97	d
		Chr7_13266188	27.23	4.30	− 1.2028	8.18	n.d
	α− carotene	Chr1_31112458	54.68	14.69	− 0.4240	28.57	d
		Chr3_4795433	9.32	8.46	0.3153	15.61	d
		Chr6_27320441	55.87	4.37	0.3204	7.92	n.d
	β− carotene	Chr1_30722692	54.34	15.66	− 0.5059	30.38	d
		Chr2_22920422	23.46	3.78	− 0.0449	6.32	n.d
		Chr3_4795433	9.32	5.92	0.2668	10.56	d
		Chr5_7281899	24.71	3.86	− 0.4798	6.24	n.d
		Chr6_4640387	29.86	4.56	− 0.6061	8.25	n.d
		Chr6_36495394	66.92	4.04	0.4557	7.41	n.d
	Lutein	Chr3_6731021	11.38	4.23	− 0.0040	7.42	n.d
		Chr5_12657999	32.41	10.23	− 0.0205	20.72	d
		Chr6_12958206	42.43	3.52	0.0095	6.61	n.d
		Chr6_20429384	49.67	3.62	0.0066	6.78	n.d
		Chr6_32850368	62.09	6.70	− 0.0509	13.39	d
	β/α− carotene ratio	Chr2_27816589	54.03	10.32	0.1376	13.01	n.d
		Chr3_23292008	32.1	5.29	− 0.1052	5.20	n.d
		Chr5_25294377	47.62	6.06	− 0.0915	7.08	n.d
		Chr6_4640387	29.86	20.82	− 0.2221	31.43	d
		Chr7_28434949	43.84	10.14	0.1431	12.68	n.d
		Chr8_29304271	61.16	4.97	0.1042	5.67	n.d
B	Visual evaluation	Chr3_5419538	18.37	6.65	− 0.9913	18.02	d

All genetic parameters were calculated by Composit Interval mapping function in QTL Cartographer ver. 2.5 (25).

^a^Population.

^b^Marker names show physical position and only one marker in the same genetic distance markers is shown. Redundant other markers without recombination are shown in Supplemental Tables S3 and S4.

^c^Log-likelihood value.

^d^Additive effect of Fs001 allele in F2 population A and Fs002 in F2 population B.

^e^Percent of phenotypic variance explained by QTL.LOD threshold to detect QTLs was determined in each trait and population (Supp. Figs. S4, S5).

^f^Detection of association analysis (Table 1 and Figs. 3, 4). QTLs detected and not detected in association analysis are indicated by d and n.d., respectively. Associations detected within 2 Mb from physical position the nearest marker were treated as identical QTL.

Interestingly, an additive effect of Fs001 allele showed the opposite effect in a visual evaluation versus color components a* and b* and α- and β-carotene contents. The QTL at a physical position around 4.8 Mb on chromosome 3 was detected for α- and β-carotene contents, and this QTL was also detected by the association analysis. In the QTL analysis, a QTL for color component a* was also detected at a physical position that is similar to the QTL for α- and β-carotene contents on chromosome 3, and this QTL was not detected by the association analysis. A QTL for visual evaluation on chromosome 5, QTLs for lutein content on chromosome 5 and 6, and a QTL for the β/α-carotene ratio on chromosome 6 were also detected by the association analysis. A QTL detected at 6.7 Mb on chromosome 3 was detected for visual evaluation, color component L*, and lutein content. A QTL detected at 13.2 Mb on chromosome 7 was detected for color components a* and b*. Compared to the association analysis, the QTL analysis detected more minor QTLs.

In F_2_ population B, only one QTL for visual evaluation was detected at physical position 5.4 Mb on chromosome 3. This QTL was also detected by the association analysis. The physical position of the nearest marker of this QTL in the QTL analysis corresponded to the peak position in the associated analysis (Tables 1, 2, Fig. 4, Suppl. Fig. S5).

### Correlations among visual evaluation, color components, and carotene contents in root of F_2_ population A

The Pearson correlation between each phenotype showed that three color components, i.e., L*, a* and b*, the α-carotene content, and the β-carotene content were highly correlated (Suppl. Table S5). The lutein content was slightly correlated with L*, a* and b* and highly correlated with the α-carotene content. As lutein is biosynthesized downstream of the α-carotene (Fig. 1), this high correlation of lutein and α-carotene is consistent with the biosynthesis pathway. The visual evaluation was not highly correlated with any other phenotypes.

### Allelic effects of QTLs detected on chromosomes 1 and 3 for the α-carotene and β-carotene contents in F_2_ population A

We examined the allelic effects of the QTLs on chromosome 1 and 3 detected by the association and QTL analyses for the α- and β-carotene contents. At the median, the carrots with AA allele on the SNP showing the highest association for α-carotene (DCARV2_CHR1_30704558) had approx. 1.5-fold higher contents of α- and β-carotene than those with GG allele (Suppl. Fig. S6a,b). Similarly, at the median, the carrots with GG allele on the SNP showing the highest association for α-carotene (DCARV2_CHR3_5849853) had approx. 1.3-fold higher contents of α-carotene and approx. 1.2-fold higher contents of β-carotene compared to those with AA allele (Suppl. Fig. S6c,d). A clear genetic interaction such as epistasis was not observed between the QTLs detected on chromosomes 1 and 3 (Suppl. Fig. S7). Together with both QTLs detected on chromosomes 1 and 3, at the median, the carrots that had alleles showing higher carotenoid content in both QTLs also had approx. 2.6-fold higher α-carotene and approx. 1.8-fold higher β-carotene contents in the epidermis and outer phloem of carrot taproot compared to those with alleles showing lower carotenoid contents in both QTLs (Suppl. Fig. S7).

### Considering of possible candidate gene for the QTL detected on chromosome 1 in F_2_ population A

The association and QTL analyses detected a QTL for visual evaluation, color components a* and b*, and α- and β-carotene contents at around 31 Mb on chromosome 1 (Tables 1, 2, Fig. 3, Suppl. Fig. S4). To explore the candidate gene of this QTL, we listed predicted genes within the confidence interval (2-LOD reduction on each side) of the physical position overlapping five traits (from 30,090,002 to 31,630,475 bp) in Supplemental Table S6. Within the confidence interval, 144 genes were predicted. Among them, DCAR_002576 encoding a photosystem II stability/assembly factor, chloroplast (HCF136), is located at 30.8 Mb. It has a function that is similar to that of a previously reported *Y* gene, which is involved in most of the carrot root color difference of white, yellow, and orange and has been hypothesized to regulate photosystem development and functional processes^10^. The genes encoding a transcription factor and unknown function were also located.

### Considering a possible candidate gene for the QTL detected on chromosome 3 and the sequence comparison between parents in F_2_ populations A and B

**Figure 5:**
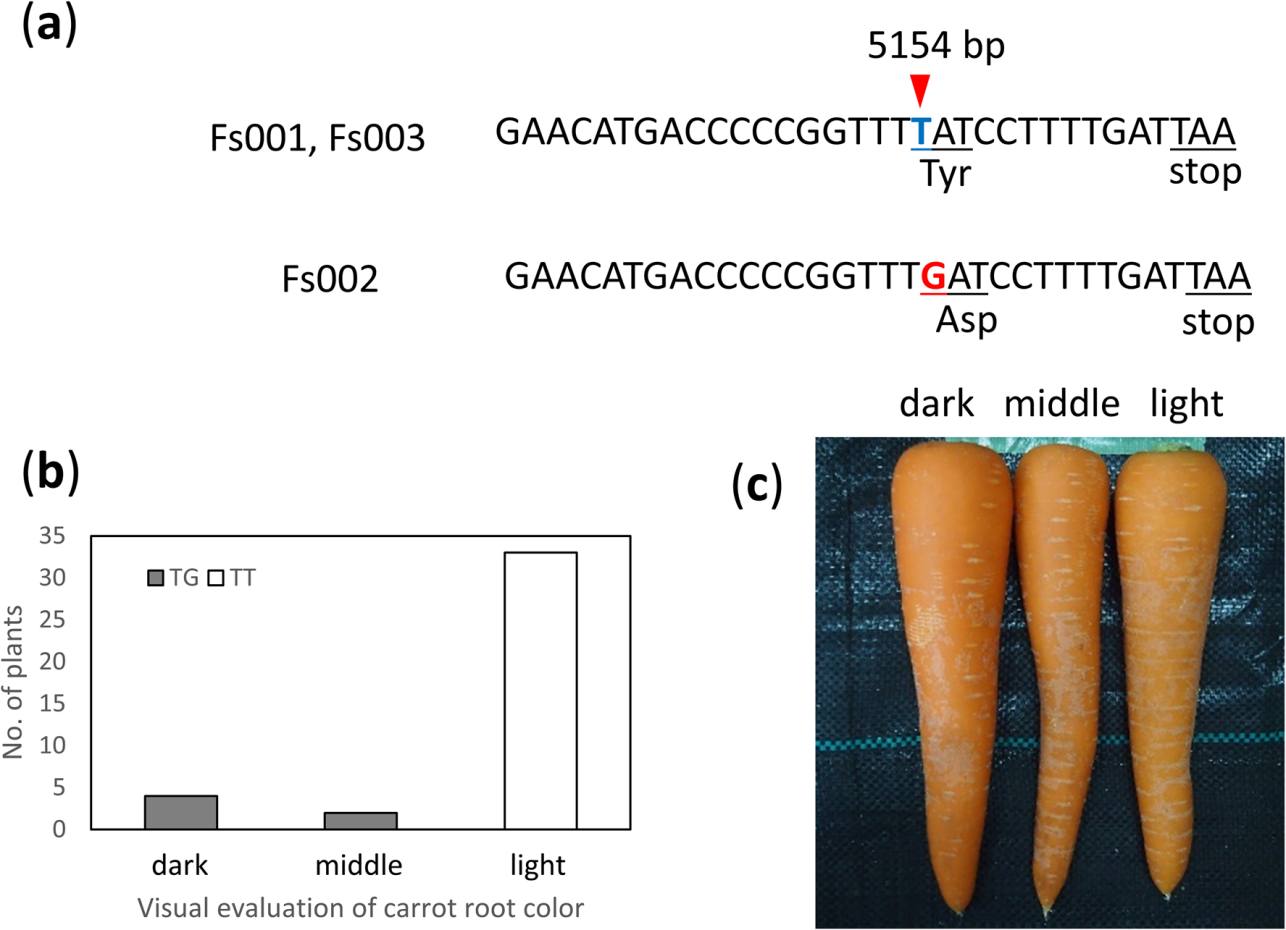
The SNP on *Or* and the examination of its effect on carrot root color in breeding line C. (**a**) The SNP detected on *Or* between parental plants in F_2_ populations A and B. The SNP causes an amino acid substitution. The *upper sequence* is identical to the reference sequence of Iorizzo et al*.*^10^, and the *lower sequence* is a new allele of *Or*. (**b**) The allelic effect of the SNP on *Or* in another breeding line at Fujii Seed. TT and TG showed TT homozygote and heterozygote of the *Or* SNP, respectively. Carrot root color was visually evaluated as three grades: dark, middle, and light orange color (b,c). All of the plants with dark or middle orange color roots showed a heterozygote for the *Or* SNP, and all plants with the slightly light orange color roots showed TT homozygote for the *Or* SNP. (**c**) Examples of carrot root color visually evaluated as three grades.

By the association and QTL analyses, the QTL was detected around the physical position at 5–6 Mb on chromosome 3 for α-carotene and β-carotene contents in F_2_ population A (Fig. 3, Suppl. Fig. S4, Tables 1, 2). Both analyses detected the QTL for visual evaluation in F_2_ population B at 5.4 Mb on chromosome 3 (Fig. 4, Suppl. Fig. S5, Tables 1, 2). Within this region, the reported *Or* gene (DCAR_009172), which affects carotenoid contents in carrot^16^, is located at 5.2 Mb. To examine the involvement of *Or*, we performed Sanger sequencing of *Or* in the parents of populations A and B. The Sanger sequencing detected a T/G SNP at the fourth codon counting from the 3’ end; it causes a non-synonymous-amino acid substitution. The SNP was detected between both parents of F_2_ populations A and B (Fig. 5a). A thymine which was identical to that in the carrot reference genome^10^ in Fs001 and Fs003 was changed to guanine in Fs002, which resulted in a change from Tyr309 in the Fs001 and Fs003 to aspartic acid in the Fs002. No other SNPs causing non-synonymous amino acid substitutions were detected on *Or* between both parents of F_2_ populations A and B.

We developed a KASP marker which could genotype the SNP on *Or*. We applied the developed KASP marker to breeding line C whose root color was segregated and that is the progeny of Fs002 (Fig. 2). The root color of breeding line C was visually evaluated into three grades (Fig. 5c). The genotype of KASP marker on *Or* was clearly correlated with the visual evaluation (Fig. 5b). All of the carrots whose root color was dark and middle orange had a heterozygote for the SNP on *Or*, and all of the carrots whose root color was light orange had a TT homozygote for the SNP. The developed KASP marker could be used for marker-assisted selection in orange root carrot breeding.

### Considering a possible candidate gene for the β/α-carotene ratio in population A, and the amino acid comparison between parents of population A

In the association and QTL analyses of F_2_ population A, the QTL for the β/α-carotene ratio was detected on chromosome 6 and showed the highest association and LOD value on the physical position at around 4.6 Mb (Tables 1 and 2, Fig. 3, Suppl. Fig. S4). Iorizzo et al.^10^ summarized the carrot orthologous and homologous candidate genes involved in the plastid 2-C-methyl-D-erythritol 4-phosphate (MEP) and carotenoid pathways in a table. According to the table, DCAR_022896 (which has a lycopene cyclase domain) is located on a physical position at 4.1 Mb on chromosome 6. Carotenoid biosynthesis bifurcates after lycopene to produce ε- and β-carotenoids by enzymatic activity of the two lycopene cyclases, lycopene ε-cyclase (LCYE) and lycopene β-cyclase (LCYB)^34^ (Fig. 1). In addition, it is known that the proportions of β-carotene and α-carotene are determined mostly by the comparative amounts and/or activities of the LCYB and LCYE enzymes^26,35–38^.

It is known that LCYB, neoxanthin synthase (NSY) (which catalyzes violaxanthin into neoxanthin), capsanthin-capsorubin synthase (CCS) (which catalyzes the conversion of antheraxanthin and violaxanthin into capsanthin and capsorubin, respectively) (Fig. 1), and chromoplast-specific lycopene β-cyclase (CYC-B) have high sequence homology and similar putative catalytic mechanisms^26,27,39,40^.

**Figure 6:**
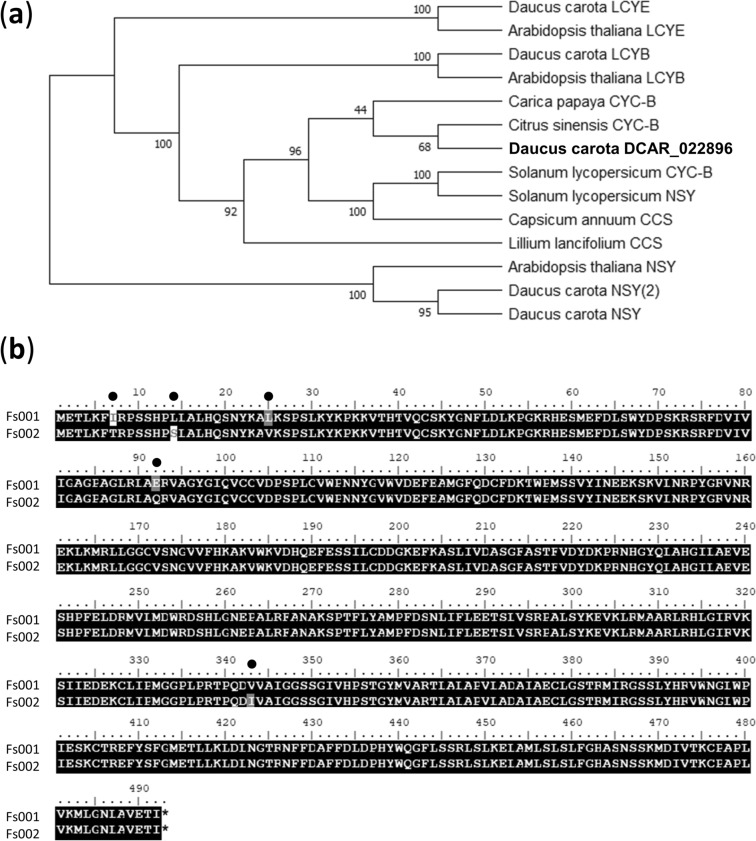
The phylogenetic tree based on the amino acid sequences of LCYE, LCYB, CYC-B, NSY, and CCS, and the amino acid substitutions between the parental plants of F_2_ population A on DCAR_022896. (**a**) The phylogenetic tree was drawn by the neighbor-joining method based on the amino acid sequences of *Daucus carota* DCAR_022896, *D.* *carota* LCYE (DCAR_028276), *Arabidopsis thaliana* LCYE (At5g57030), *D.* *carota* LCYB (DCAR_020544), *A.* *thaliana* LCYB (At3g10230), *Solanum lycopersicum* CYC-B, *Carica papaya* CYC-B (evm.model.supercontig_195.16), *Citrus sinensis* CYC-B (orange1.1g010693m.g), *D.* *carota* NSY (DCAR_017191), *D.* *carota* NSY(2) (DCAR_025914), *A.* *thaliana* NSY (At1g67080), *S.* *lycopersicum* NSY (CAB93342.1), *Capsicum annuum* CCS (Q42435.1), and *Lillium lancifolium* CCS (JF304153). Values at the nodes indicate the percentage consensus support as calculated using a bootstrapping test with 1,000 replications. (**b**) Five amino acid substitutions (black filled circle) were detected between seed (Fs001) and pollen (Fs002) parents of F_2_ population A on DCAR_022896. Similar substitutions are shown in *gray* background.

Our phylogenic analysis of DCAR_022896, LCYE, LCYB, NSY, CCS, and CYC-B in carrot and *Arabidopsis* as well as *Solanum lycopersicum*, *Carica papaya*, *Citrus sinensis*, *Capsicum annuum*, and *Lillium lancifolium* showed that DCAR_022896 belonged to the same clade as CYC-B in *C.* *sinensis* and *C.* *papaya* (Fig. 6a). At the amino acid level, DCAR_022896 had 76.9% identity to CYC-B in *C. sinensis* and 62.1% to CYC-B in *C. papaya*. CYC-B is a *LCYB*, and it converts lycopene to β-carotene in chromoplasts, where carotenoids are accumulated^41,42^, in a specific manner^26^ (Fig. 1). Moreover, our BLAST search of primers for the reported *LCYB2* in carrot showed that *CYC-B* (DCAR_022896) in the present study is identical to *LCYB2*^5,6,43,44^. We thus presume that DCAR_022896 might be a possible candidate gene for the β/α-carotene ratio, and we compared the amino acid sequences between the parents of F_2_ population A by Sanger sequencing. The amino acid comparison revealed five amino acid substitutions between the parents of F_2_ population A (Fig. 6b). These results suggested the possibility that *CYC-B* might be a candidate gene for the QTL and the involvement of CYC-B in the β/α-carotene ratio in carrot root.

## Discussion

Our association and QTL analyses using the two F_2_ populations derived from orange root carrots detected 21 and 32 QTLs for carrot root color traits(Figs. 3, 4, Suppl. Figs. S4, S5, Tables 1, 2). The QTL detected at around 31 Mb on chromosome 1 was detected by both analyses and showed high -log10P and LOD values; this explained much of the phenotypic variance for visual evaluation, color components a* and b*, and α- and β-carotene contents. The G allele at the physical position at 30,704,558 bp on chromosome 1 was dominant to A allele for α- and β-carotene contents (Supp. Fig. S6). To breed and select carrots that have higher carotenoid contents, the selection of a homozygous AA allele for this locus would be necessary. The quantitative differences in root color and carotenoid contents are affected by environmental conditions such as temperature and the field and light conditions after harvest. A complete selection for root color and carotenoid contents by appearance is thus very difficult. DNA markers of the QTL at around 31 Mb on chromosome 1 as well as the QTL on the chromosome 3 would be helpful for orange carrot breeding (Suppl. Figs. S6, S7).

There are no annotated genes for MEP and carotenoid pathways within 5 Mb from the physical position of 31 Mb on chromosome 1 where the highest association and LOD score was detected^10^. However, DCAR_002576 (the gene encoding photosystem II stability/assembly factor) is located at 30.8 Mb, and this function is similar to that of *Y*, accounting for the color difference of white, yellow and orange in carrot root; both genes have a role in the photosystem^10,45^. In the storage root of sweet potato, several genes involved in plastid biosynthesis including photosystem II are differentially expressed between white mutant and β-carotene-accumulating orange sweet potato cultivars^46^. It might be likely that polymorphism of DCAR_002576 causes a quantitative difference in carotenoid contents and affects root color, although further analyses are needed to explore this possibility.

A major QTL for lutein content was detected in F_2_ population A on chromosome 5 (Fig. 3, Suppl. Fig. S4, Tables 1, 2), whereas there are no predicted genes annotated for MEP and carotenoid pathways^10^ or for chromatin-modifying histone methyltransferase, *SDG8* (*CCR1*), which affects the lutein content in leaves^47^ around this locus except for *neoxanthin synthase* (*NSY*). The *NSY* gene is located approx. 1.1 Mb away from the physical position of the highest association for lutein content. NSY has a role downstream of another branch which does not include lutein in carotenoid biosynthesis (Fig. 1), and no feedback regulation between NSY and lutein content has been reported. However, we cannot exclude the possibility that the mutation of the *NSY* of another branch affects the flow rate of each branch, resulting in an effect on the lutein content. Further analyses such as a map-based strategy is necessary to narrow down the candidate regions and identify candidate genes for QTLs on chromosome 1 for several color phenotypes and chromosome 5 for lutein content, and for the other significant QTLs revealed in this study.

The QTL analysis detected QTL at 6.7 Mb on chromosome 3 for visual evaluation, color component L*, and lutein content. The physical position of the QTL is similar to those of the QTL detected at 4.1 Mb for color component a* and the QTL detected at 4.8 Mb for α- and β-carotene contents. Further analysis is necessary to determine whether or not these QTLs are identical. The QTLs exerting a large effect were detected by both the association and QTL analyses. However, QTLs exerting a small effect would not be detected by both analyses because of false-positive and false-negative detections. The 11 QTLs detected by both analyses in this study are more reliable.

The Pearson correlation showed no high correlation between visual color evaluation and other phenotypes (Suppl. Table S2). Experienced breeders evaluate root color comprehensively including the gloss and texture of the carrot surface, and thus the detected associations only for visual evaluation might be associated with these phenotypes.

Carrot *Or* was recently identified and is associated with the carotenoid presence in carrot root^16^. The proximity of physical positions and function of *Or* suggests that QTL detected at around 5–6 Mb on chromosome 3 were possibly caused by *Or*. An SNP causing a non-synonymous mutation was detected in the present study between the parents of F_2_ populations A and B by Sanger sequencing (Fig. 5a). We could not compare the sequences of the promotor region at the upstream of *Or* in the parental lines, and we therefore cannot exclude the possibility that polymorphism(s) at the promoter region causes the phenotypic difference. As breeding line C was derived from Fs002 (Fig. 2), a dark orange allele would be derived from the Fs002.

The association and QTL analyses in F_2_ population A revealed the QTL for the β/α-carotene ratio on chromosome 6; *CYC-B* is located on the QTL region (Fig. 3h, Suppl. Fig. S4h, Tables 1, 2). In several model plants such as *Arabidopsis thaliana*^48^, rice (*Oryza sativa*)^49^, and maize (*Zea mays*)^35,50^, LCYB is encoded by a single gene. However, LCYB is encoded by two genes in some plant species that accumulate high levels of carotenoids in non-photosynthetic organs, such as fruits and flowers^44^. These genes are differentially expressed in photosynthetic and non-photosynthetic organs, and genes that are expressed in non-photosynthetic organs were named *CYC-B*. As named, *CYC-B* is a chromoplast-specific lycopene β-cyclase.

Carrot has two LCYBs: LCYB1 and LCYB2^3^. Our present phylogenetic analysis demonstrated that carrot LCYB1 (LCYB) and LCYB2 (CYC-B) belong to LCYB and CYC-B clades, respectively (Fig. 6a). The *CYC-B* was first reported in tomato (*Solanum lycopersicum*) as a fruit- and flower-specific lycopene β-cyclase^26^ and has also been reported to be responsible for fruit color in papaya (*Carica papaya*)^28^ and citrus (*Citrus sinensis*) and for high lycopene accumulation in red grapefruits^29^. In carrot, unlike plants that have organ-specific LCYBs, *LCYB1* is expressed in both leaves and root, and the transcript level of *LCYB1* increases as the carotenoid content increases during root development^44,51^. Since the association and QTL analyses detected a QTL around the *CYC-B* region in this study, we speculate that in carotenoid-accumulating carrot root, in addition to the LCYB (LCYB1), CYC-B (LCYB2) might also have a role in carotenoid biosynthesis. Further functional analyses such as an expression study of *CYC-B* (*LCYB2*) in several organs and developmental stages and an investigation of the subcellular location of CYC-B (LCYB2) in carrot are necessary to clarify the involvement of CYC-B in the carotenoid contents of carrot taproot and the functions of the two LCYBs in carrot.

Visual appearance traits are important targets in carrot breeding in Japan, and the 'best bright orange color' is selected based on a comparison of minute color differences as shown in Fig. 5c. The present study provides the first results of association and QTL analyses for carrot root color for the selection of bright orange color in orange root populations. The developed KASP marker on *Or* as well as the SNPs showing significant associations will contribute to orange carrot breeding.

## Supplementary Information

Supplementary Tables.

Supplementary Figures.

## Data Availability

Nucleotide sequence data for the ddRADseq in F_2_ population A and B is available in the DDBJ Sequence Read Archive under accession numbers from DRA012848 to DRA012853.
