# Supplementary material for: Identification of QTLs for root color and carotenoid contents in Japanese orange carrot F2 populations
Source: Sci Rep. 2022 May 16;12:8063. doi: 10.1038/s41598-022-11544-7 (PMC9110420; doi:10.1038/s41598-022-11544-7)
Supplement: Supplementary file 2 — Supplementary Figures. [file 41598_2022_11544_MOESM2_ESM.pdf]

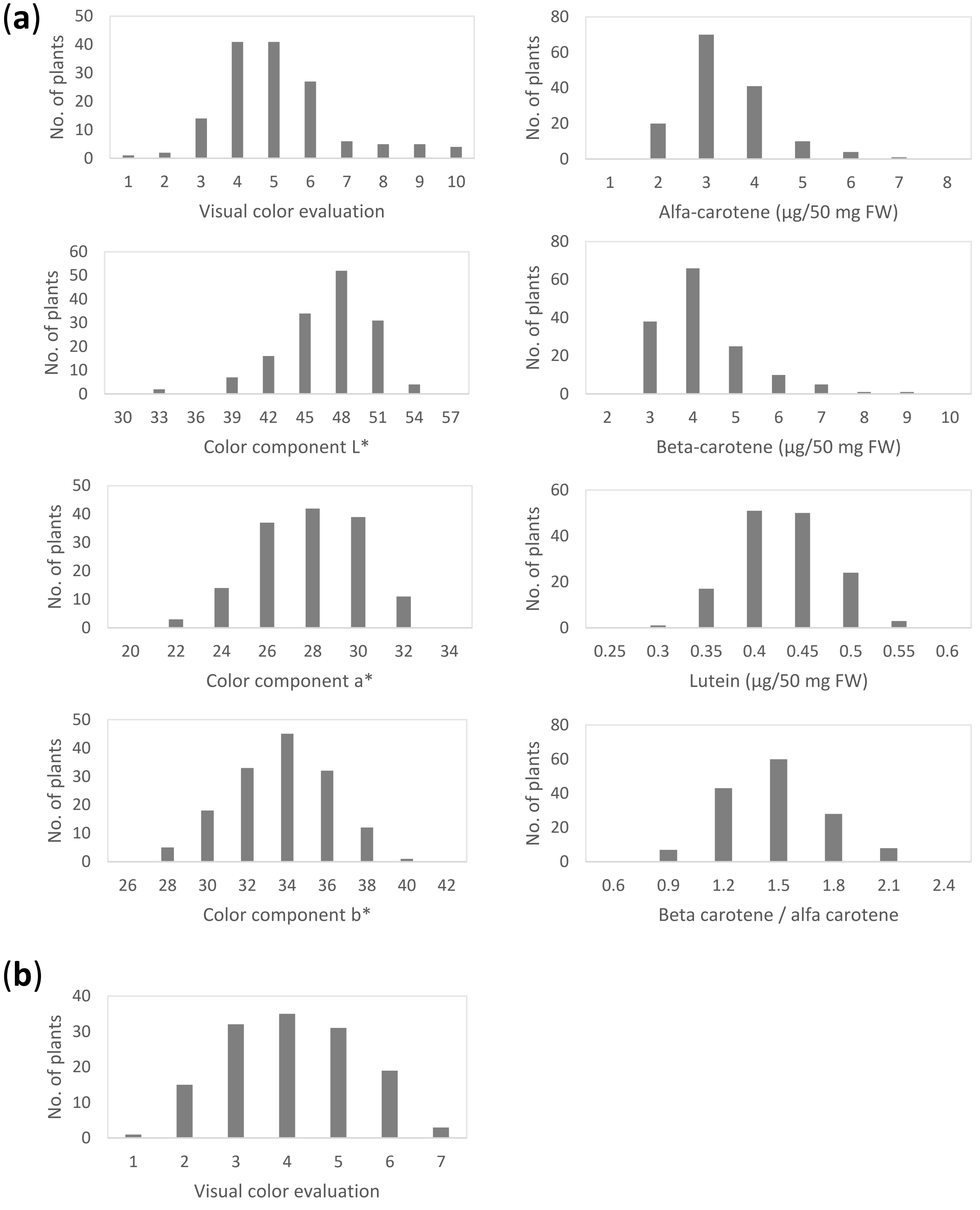


**Suppl. Fig. S1.** The frequency distributions of carrot root color in $F_2$ population A **(a)** and $F_2$ population B **(b).** In $F_2$ population A, roots were visually evaluated, and color components (L*, a*, and b*) and α- and β-carotene contents were examined (a). In $F_2$ population B, roots were only visually evaluated (b). In the visual color evaluation, root color was categorized in ten grades (1: light orange to 10: dark orange) in $F_2$ population A (a) and seven grades (1: light to 7: dark orange) in $F_2$ population B (b).

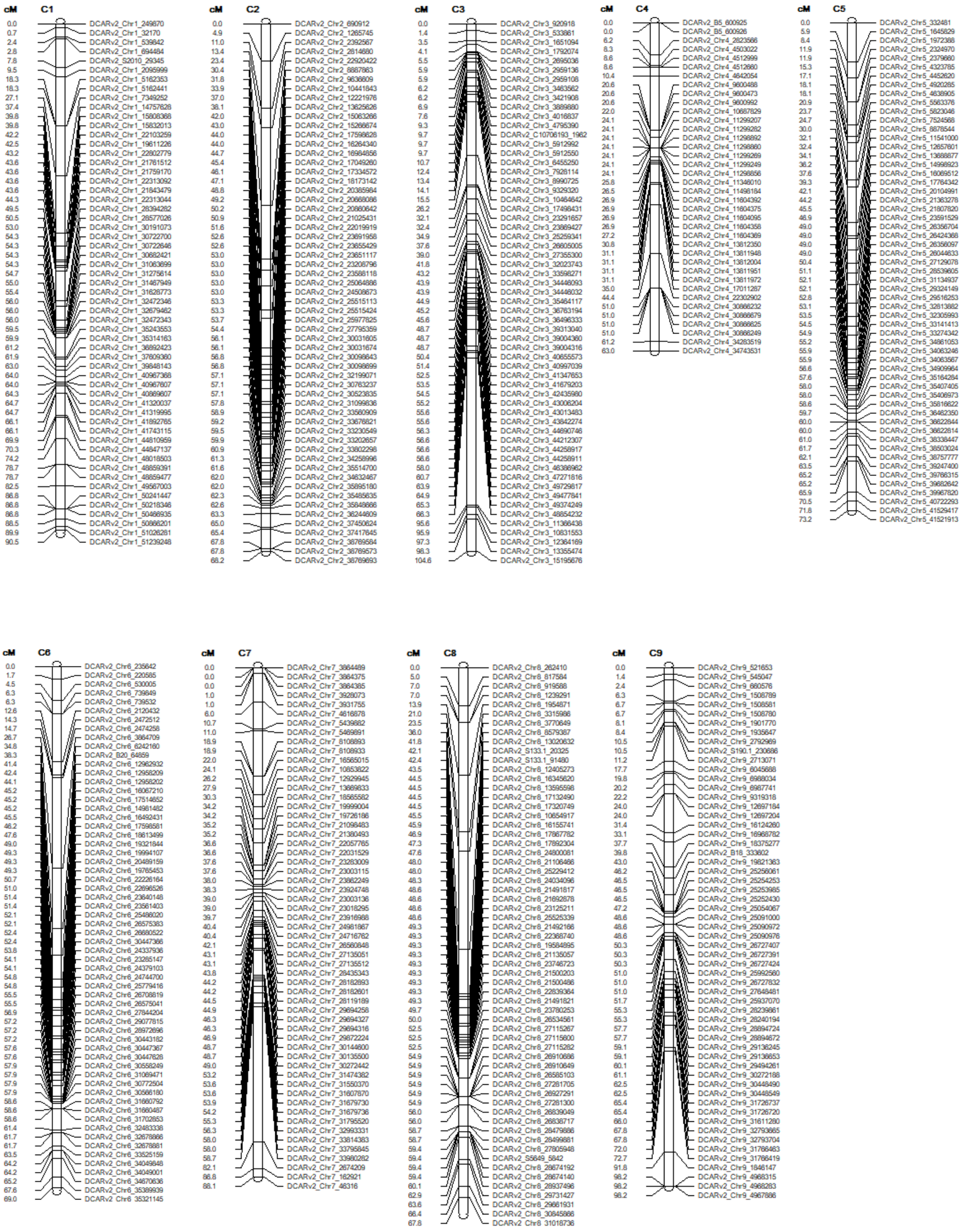

**Suppl. Fig. S2** Suppl. Fig. S2. Schematic image of the linkage map of $F_2$ population A. Not all of the markers are shown. Markers on this image were selected automatically by QTL Cartographer ver. 2.5 (25). Most of the redundant markers were excluded in the map. All markers and genetic positions in this map are listed in Supplementary Table S3.

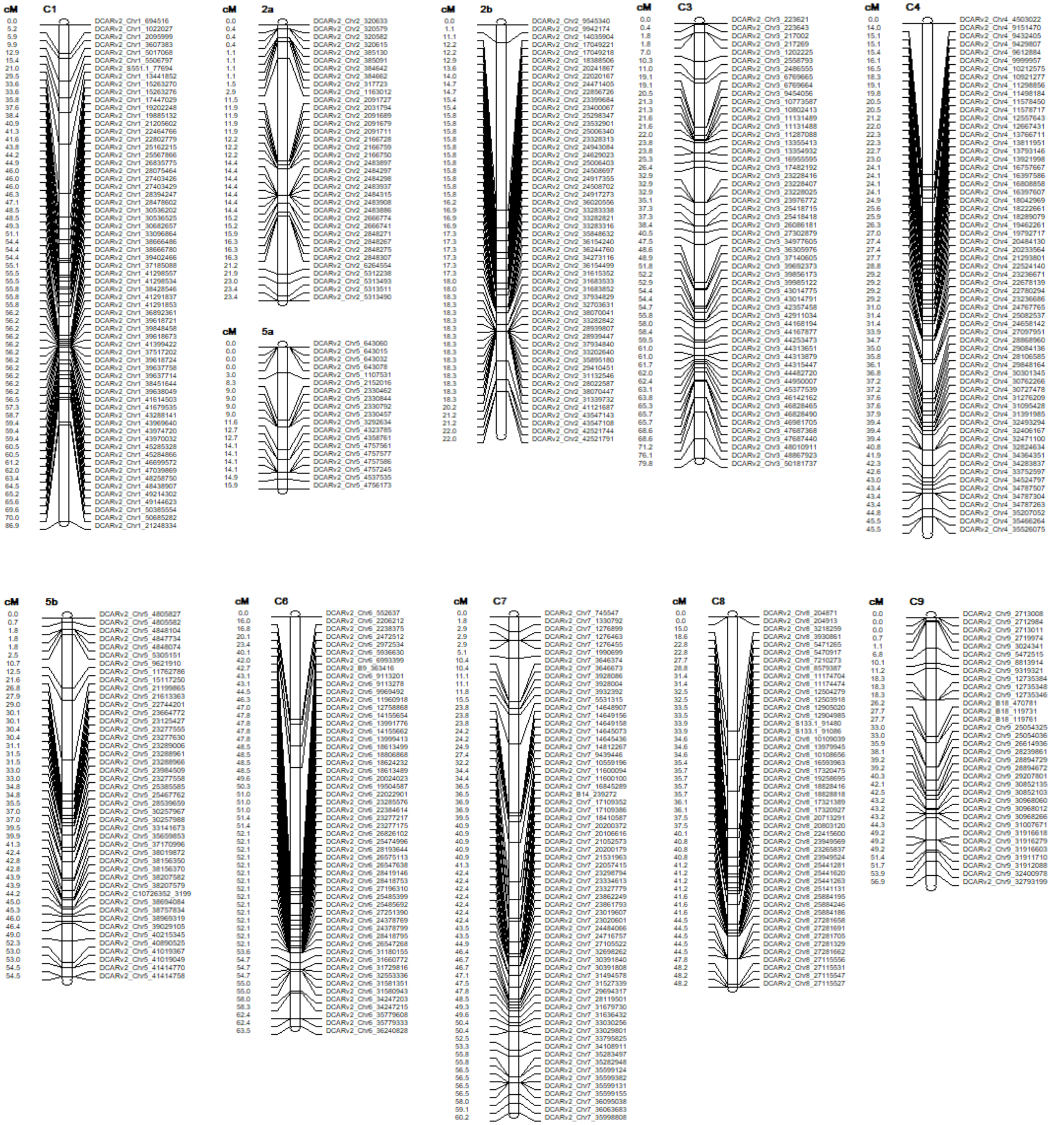


**Suppl. Fig. S3.** Schematic image of the linkage map of $F_2$ population B. Not all of the markers are shown. Markers on this image were selected automatically by QTL Cartographer ver. 2.5 (25). Most of the redundant markers were excluded in the map. All markers and genetic positions in this map are listed in Supplementary Table S4.

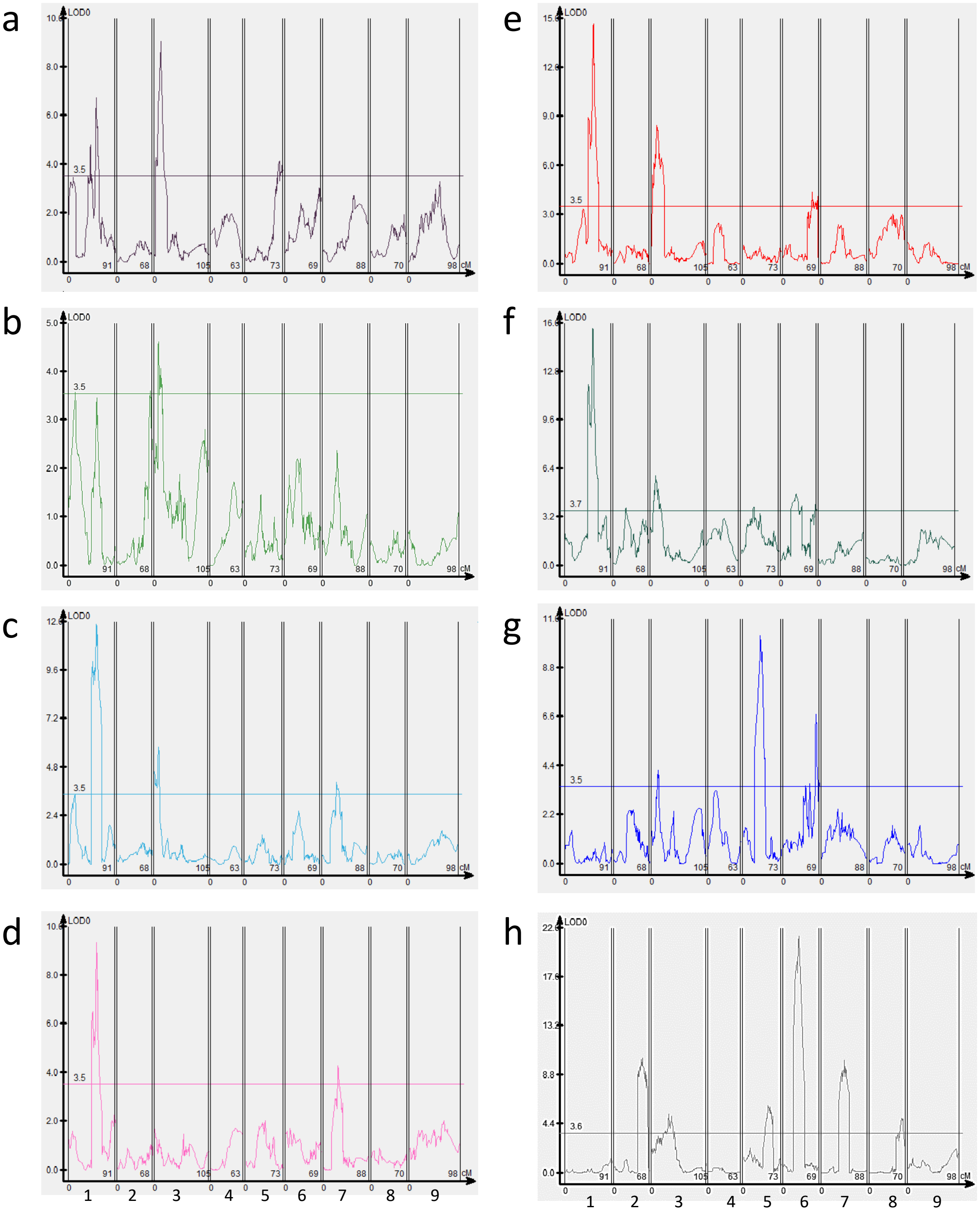

**Suppl. Fig. S4.** Log likelihood (LOD) in the QTL analysis of $F_2$ population A for nine carrot chromosomes. A threshold to declare a putative QTL is indicated by the *horizontal line*. The threshold values for LOD scores were determined from 1,000 permutation tests. **a:** Visual evaluation, **b:** L*, **c:** a*, **d:** b*, **e:** α-carotene content, **f:** β-carotene content, **g:** lutein content, **h:** the β/α-carotene ratio.

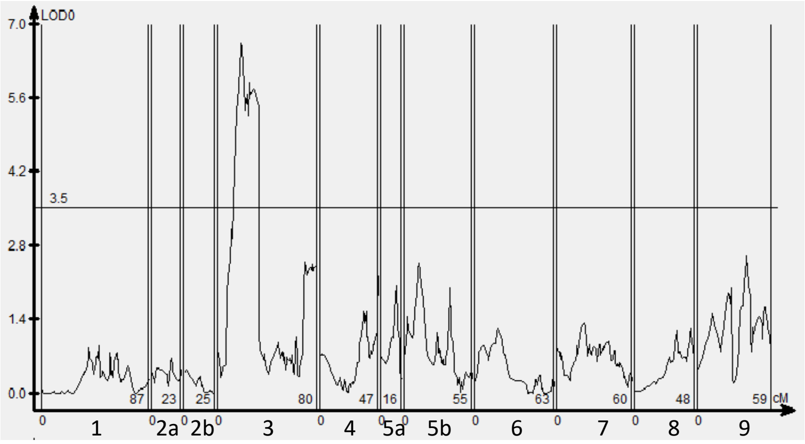


**Suppl. Fig. S5.** QTL for the visual evaluation of carrot taproot color in $F_2$ population B. LOD for carrot chromosomes and linkage groups. A threshold to declare a putative QTL is indicated by the *horizontal line*. The threshold values for LOD score were determined from 1,000 permutation tests.

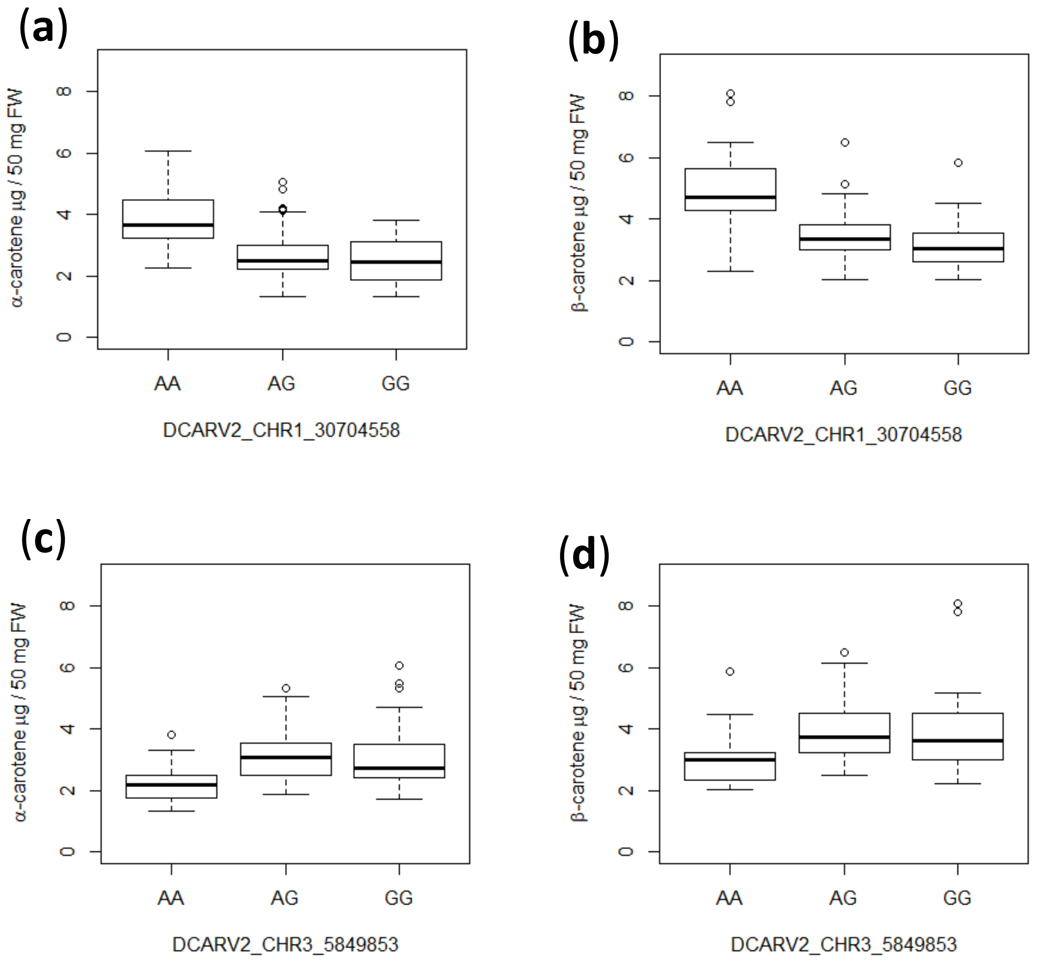


**Suppl. Fig. S6.** Allelic effects of associations detected by the GWAS on chromosome 1 (**a**, **b**) and chromosome 3 (**c**, **d**) for the α-carotene (**a**, **c**) and β-carotene (**b**, **d**) content in carrot root surface. Carotene contents were box-plotted by the SNP showing the highest –log10P in the GWAS for α-carotene content. The numbers of plants were 30, 81, and 34 for the AA, AG, and GG SNPs respectively in panels **a** and **b**, and 40, 70, 35 for the AA, AG, and GG SNPs respectively in panels **c** and **d**.

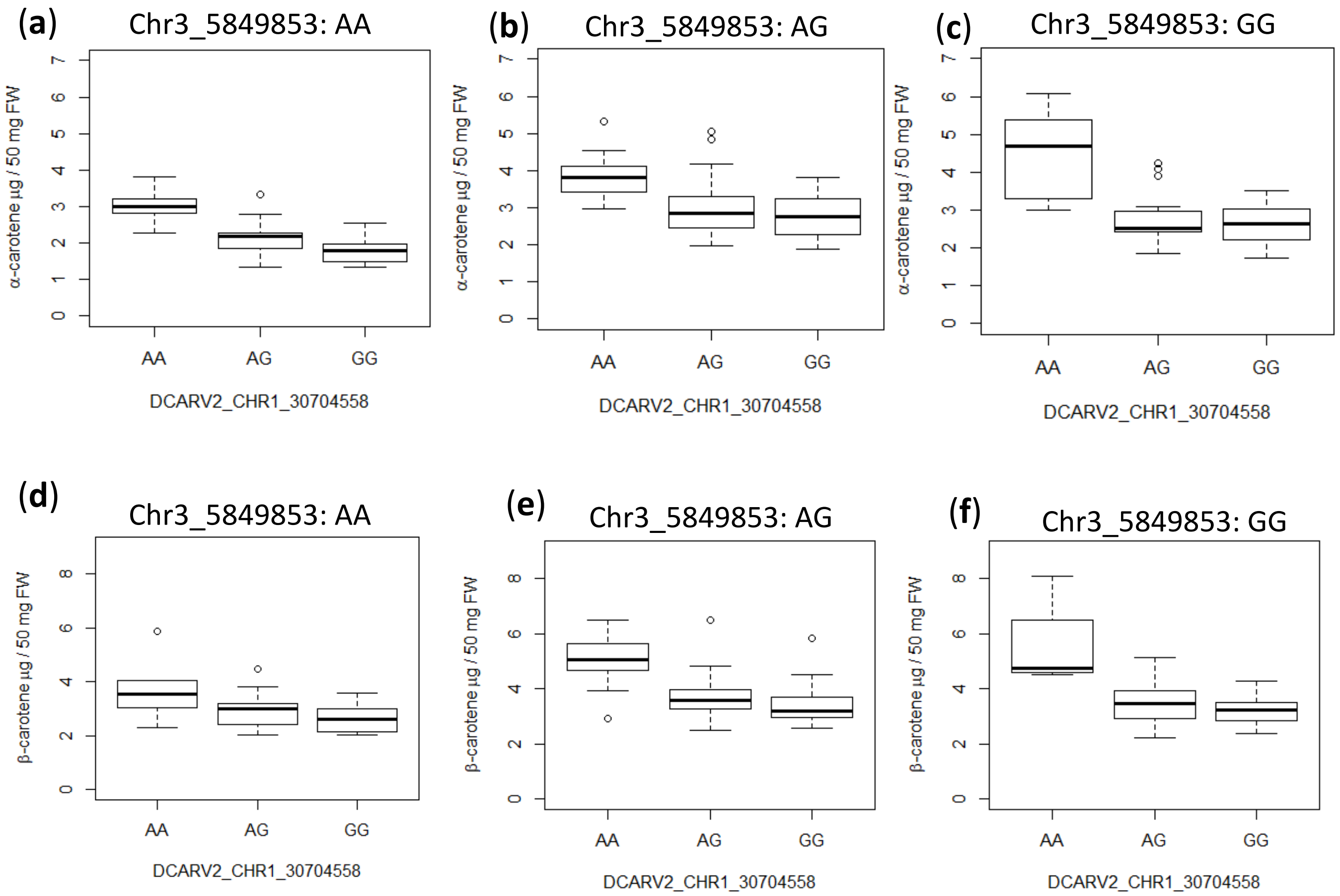


**Suppl. Fig. S7.** Examination of allelic interactions between associations detected by GWAS on chromosomes 1 and 3 for the α-carotene **(a–c)** and β-carotene **(d–f)** content in carrot root surface. Carotene contents were box-plotted by the SNP showing the highest –log10P in the GWAS for the α-carotene content on the chromosomes 1 and 3. The SNPs of chromosome 3 at physical position 5,849,853 were AA **(a, d)**, AG **(b, e)**, and GG **(c, f)**.
